# Telomere-to-telomere genome and resequencing of 231 individuals reveal evolution, genomic footprints in Asian icefish, *Protosalanx chinensis*

**DOI:** 10.1093/gigascience/giaf067

**Published:** 2025-07-17

**Authors:** Yanfeng Zhou, Chenhe Wang, Binhu Wang, Dongpo Xu, Xizhao Zhang, You Ge, Shulun Jiang, Fujiang Tang, Chunhai Chen, Xuemei Li, Jianbo Jian, Yang You

**Affiliations:** Key Laboratory of Freshwater Fisheries and Germplasm Resources Utilization, Ministry of Agriculture and Rural Affairs, Freshwater Fisheries Research Center, Chinese Academy of Fishery Sciences, Wuxi 214081, China; Wuxi Fisheries College, Nanjing Agricultural University, Wuxi 214081, China; BGI Genomics, Shenzhen 518083, China; Key Laboratory of Freshwater Fisheries and Germplasm Resources Utilization, Ministry of Agriculture and Rural Affairs, Freshwater Fisheries Research Center, Chinese Academy of Fishery Sciences, Wuxi 214081, China; Key Laboratory of Freshwater Fisheries and Germplasm Resources Utilization, Ministry of Agriculture and Rural Affairs, Freshwater Fisheries Research Center, Chinese Academy of Fishery Sciences, Wuxi 214081, China; Key Laboratory of Freshwater Fisheries and Germplasm Resources Utilization, Ministry of Agriculture and Rural Affairs, Freshwater Fisheries Research Center, Chinese Academy of Fishery Sciences, Wuxi 214081, China; Key Laboratory of Freshwater Fisheries and Germplasm Resources Utilization, Ministry of Agriculture and Rural Affairs, Freshwater Fisheries Research Center, Chinese Academy of Fishery Sciences, Wuxi 214081, China; Heilongjiang River Fisheries Research Institute, Chinese Academy of Fishery Sciences, Harbin 1150070, China; BGI Genomics, Shenzhen 518083, China; Yangtze River Fisheries Research Institute, Chinese Academy of Fishery Sciences, Wuhan 430223, China; BGI Genomics, Shenzhen 518083, China; Guangdong Provincial Key Laboratory of Marine Biotechnology, Shantou University, Shantou 515063, China; Key Laboratory of Freshwater Fisheries and Germplasm Resources Utilization, Ministry of Agriculture and Rural Affairs, Freshwater Fisheries Research Center, Chinese Academy of Fishery Sciences, Wuxi 214081, China

**Keywords:** telomere-to-telomere, genome assembly, protosalanx chinensis, evolution, comparative genomics

## Abstract

The Asian icefish, *Protosalanx chinensis*, has undergone extensive colonization in various waters across China for decades due to its ecological and physiological significance as well as its economic importance in the fishery resource. Here, we decoded a telomereto-telomere (T2T) genome for *P. chinensis* combining PacBio HiFi long reads and ultra-long ONT (nanopore) reads and Hi-C data. The telomere was identified in both ends of the contig/chromosome. The expanded gene associated with circadian entrainment suggests that *P. chinensis* may exhibit a high sensitivity to photoperiod. The contracted genes’ immune-related families and DNA repair associated with positive selection in *P. chinensis* suggested the selection pressure during adaptive evolution. The population genetic analysis reported the genetic diversity and genomic footprints in 231 individuals from 7 different locations. The introduced highest alkalinity population (HRCL) exhibited higher values of inbreeding coefficients and clustered different from other groups suggested local environmental adaptation. Thus, the T2T genome and genetic variation can be valuable resources for genomic footprints in *P. chinensis*, shedding light on its evolution, comparative genomics, and the genetic differences between natural and introduced populations.

## Introduction

*Protosalanx chinensis* (NCBI: txid240822; marinespecies.org: taxname: 315354), belonging to the family Salangidae, is a diminutive annual fish species indigenous to East Asia, characterized by its translucent body, scaleless skin and cartilaginous bones [1–4]. The largest population of *P. chinensis* is found in China. There are two kinds of natural distribution of *P. chinensis* included inshore migration and landbound. In China, the migratory populations predominantly inhabit the coastal estuaries of the Bohai Sea, Yellow Sea, and East China Sea [5]. The landbound populations are distributed in seagoing rivers and adjacent lakes, with the highest abundance observed along the Yangtze River basin, including Taihu Lake, Hongze Lake, and Chaohu Lake [1, 5, 6]. Due to the significant economic value of *P. chinensis*, since the mid-1980s, there has been extensive colonization of *P. chinensis* populations in various waters across China, particularly the expansion in northern China [7]. The deliberately introduced location environments provided distinct habitats for *P. chinensis*, characterized by variations in water quality, temperature, salinity, and community structure [6, 8]. The selection pressure exerted by the diverse habitat conditions constitutes a crucial factor in facilitating the local adaptation of *P. chinensis* [9]. Some introduced populations in northern China exhibit substantial genetic differentiation compared to native populations, while maintaining a commendable level of genetic diversity [10]. However, post-introduction failure had been widely reported due to overfishing, pollution, food shortages and so on [1, 6, 11]. In addition to the influence of external environmental factors, it is imperative to consider the genetic bottleneck encountered by the introduced population and the subsequent reduction in genetic diversity [6].

From the perspective of ecological conservation and fishery resource utilization, it is imperative to comprehend the fundamental structure of the spatial distribution pattern of their intricate habitat environment and historical introduction. Although previous studies utilizing mitochondrial genes, microsatellites, and whole genome sequencing have initially demonstrated genetic differentiation among different ecotypes of *P. chinensis* and inferred changes in genetic diversity within migratory populations [1, 5, 6]. At present, the previously studies of *P. chinensis* populations have not comprehensively addressed the full range of habitats inhabited by this species. In a broader sampling range, it is imperative to investigate the genetic status of *P. chinensis* populations, particularly focusing on enhancing research in the primary introduced region (northeast China).

The comprehensive understanding of the genetic mechanism underlying *P. chinensis* necessitates the resources of reference genome. In 2017, the first draft genome (contig N50: 17.2 Kb, Scaffold N50:1.16 Mb) was finished based on short-read sequencing Illumina platform without chromosome-level [2]. A higher quality genome of *P. chinensis* was reported, exhibiting a contig N50 of 103 kb and scaffold N50 of 5.1 Mb, achieved through PacBio long-read sequencing [3]. The PacBio HiFi long-read and Hi-C data were combined to assemble a chromosome-level genome, resulting in a contig N50 of 0.53 Mb and scaffold N50 of 14.52 Mb [12]. However, the quality of the three previously reported genomes still falls significantly short when compared to the Telomere-to-telomere (T2T) genome. Combining the ultra-long nanopore sequencing data, the T2T genome has been successfully assembled in humans [13], while gap-free genome assemblies have been achieved in fish [14, 15]. To more comprehensive understanding the reference information, in here, a T2T genome of *P. chinensis* was successfully assembled combining PacBio HiFi, ultra-long nanopore and Hi-C data.

Population genomics is an effective technology can elucidate demographic history, population structure, inbreeding and selective processes [16]. Although the P. chinensis population, encompassing both native and numerous geographic populations in China, has received limited attention in terms of deciphering phylogenetic relationships and population structure. The introduced population has also never been documented, resulting in the dissemination of ambiguous information. The evolutionary fate of introduced populations was characterized through a previous study involving 70 individuals from 7 geographic populations [6]. In this study, a T2T genome was assembled to facilitate investigations into its evolutionary dynamics and comparative genomics. A larger number of samples (a total of 231 individuals from 7 geographic sites) were collected for whole genome sequencing to investigate the genetic diversity, phylogenetic relationships, and adaptation.

## Results

### Sequencing and *De novo* assembly

**Figure 1:**
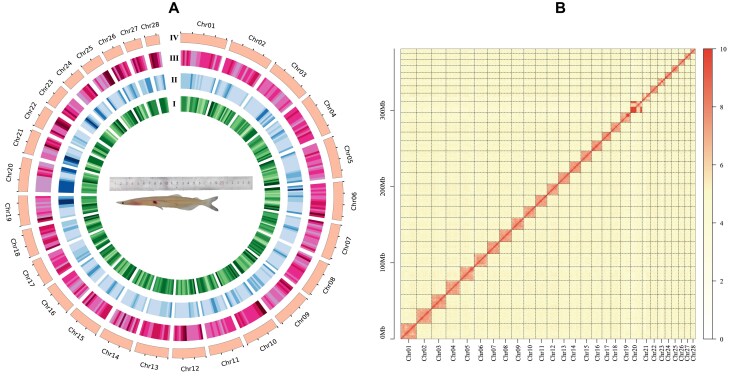
Genome features of *Protosalanx chinensis* genome. A. Circos plot showing the characterization of *Protosalanx chinensis* genome. From inside to outside: I: GC content in non-overlapping 1Mb windows; II: percentage of repeat sequence in 1 Mb sliding windows; III: gene density in 1-Mb sliding windows; IV: The length of pseudo-chromosome (scale: 5Mb). B. Heat map displaying Hi-C interactions of *Protosalanx chinensis* genome.

To generate Telomere-to-telomere genome for *P. chinensis*, PacBio HiFi long reads, and ultra-long ONT (nanopore) reads and Hi-C data were sequenced, respectively. After discarding the low-quality reads, a total of approximately 24.75 Gb (61.88 ×) PacBio HiFi data, 32.31 Gb (80.78 ×) ONT data and 107.01 Gb (2267.53×) Hi-C data were used for *de novo* assembly (Supplementary Table S1). The *P. chinensis* genome was assembled using HiFi reads, then the Hi-C data successfully assigned the assembled sequences to the 28 chromosomes (Fig. 1A and B). The T2T assembled genome size of *P. chinensis* was approximately 380.76 Mb after ultra-long ONT gap filling, with contig values ranging from 6.2 Mb to 20.52 Mb and consisting of 28 representative contigs corresponding to 28 chromosomes (Table 1). The telomere repeat monomer (AACCCT) allows for the identification of both telomeres in each contig/chromosome (Supplementary Table S2). The newly sequenced genome had greatly improved the contiguity, accuracy, and completeness compared to the previously published *P. chinensis* genome. The accuracy of the T2T assembly was evaluated using Merqury with a 21-mer, resulting in a quality value (QV) score of 40.7 and an associated accuracy of 99.9915%. The comparison of 1929 contigs with only 28 in the new genomes revealed a total of 392 new genes that were absent from the previous genome. While no gaps were found in the new genome, there are still 1919 gaps present in the previous one (Table 1). The new T2T genome was subjected to BUSCO V5 analysis using the actinopterygii_odb10 gene set, which revealed that a total of 89.4% of genes were complete. Among these, 87.3% were single copies while 2.1% showed duplication.

**Table 1:** The comparison of genome assembly between the new genome and previously genome in *P. chinensis*.

	*P. chinensis* (This study)				*P. chinensis* [12]		
ID	Length (bp)	Contig Number	Gap Number	New Assembled Gene	Length (bp)	Contig Number	Gap Number
Chr01	20,517,911	1	0	12	20,420,276	52	51
Chr02	19,251,107	1	0	11	18,886,921	73	72
Chr03	18,536,346	1	0	9	18,496,308	77	76
Chr04	18,409,333	1	0	17	17,712,959	78	77
Chr05	18,095,643	1	0	11	18,082,065	79	78
Chr06	16,963,780	1	0	16	16,854,175	84	83
Chr07	15,821,133	1	0	13	15,801,667	63	62
Chr08	15,757,408	1	0	7	15,704,969	57	56
Chr09	15,689,130	1	0	12	15,247,643	89	88
Chr10	15,058,275	1	0	17	14,596,036	66	65
Chr11	14,852,930	1	0	9	14,559,537	74	73
Chr12	14,594,547	1	0	12	14,524,669	59	58
Chr13	14,770,876	1	0	9	14,516,431	82	81
Chr14	14,458,632	1	0	4	14,365,668	63	62
Chr15	14,021,098	1	0	9	13,763,165	70	69
Chr16	13,244,385	1	0	14	12,769,249	75	74
Chr17	11,532,641	1	0	20	12,140,554	71	70
Chr18	12,206,690	1	0	17	11,537,031	64	63
Chr19	13,137,870	1	0	16	11,457,059	70	69
Chr20	14,969,277	1	0	14	10,973,078	61	60
Chr21	10,736,073	1	0	20	10,382,235	73	72
Chr22	9,479,737	1	0	12	8,471,217	66	65
Chr23	9,377,620	1	0	13	8,835,516	62	61
Chr24	8,444,670	1	0	9	8,622,590	77	76
Chr25	9,021,203	1	0	42	8,599,918	57	56
Chr26	8,147,314	1	0	29	8,044,071	62	61
Chr27	7,457,647	1	0	9	7,821,795	71	70
Chr28	6,204,715	1	0	9	7,762,152	54	53
Total	380,757,991	28	0	392	370,948,954	1,929	1,901

#"New Assembled Gene" indicated the gene located at the region of PAV region compared to the *P. chinensis* (Zhou et al. 2023) genome assembly.

### Genome annotation

After conducting repeat identification, a total of 137.79 Mb (35.9% of the assembled genome) transposable elements (TEs) were identified, with Retro/LINE accounting for the highest proportion (Supplementary Tables S3 and S4 and Supplementary Fig. S1). The gene annotation predicted a total of 21,073 protein-coding genes in the *P. chinensis* genome, with an average gene length of 8,515 (Supplementary Table S5). The average length of exon and the average length of intron was 174 bp and 785 bp, respectively (Supplementary Table S5). The protein-coding genes in *P. chinensis* genomes were supported by at least one evidence, exhibiting a CDS overlap ratio greater than 50% and 80% with a confidence level of 100% and 99.59%, respectively (Supplementary Table S6). In total, 20,188 (95.8%) gene models in the *P. chinensis* genome were annotated in at least one of the seven databases (Supplementary Table S7), whereas 73.59% (15,507) of *P. chinensis* genes and were annotated in five functional databases (NR, KEGG, KOG, SwissProt, and InterPro) (Supplementary Table S7 and Supplementary Fig. S2). The comparison of gene features revealed that the length of exons and coding sequences (CDS) was conserved among the related fish species. However, there were significant differences in mRNA and intron lengths (Supplementary Fig. S3).

### Comparative genomic analysis

**Figure 2:**
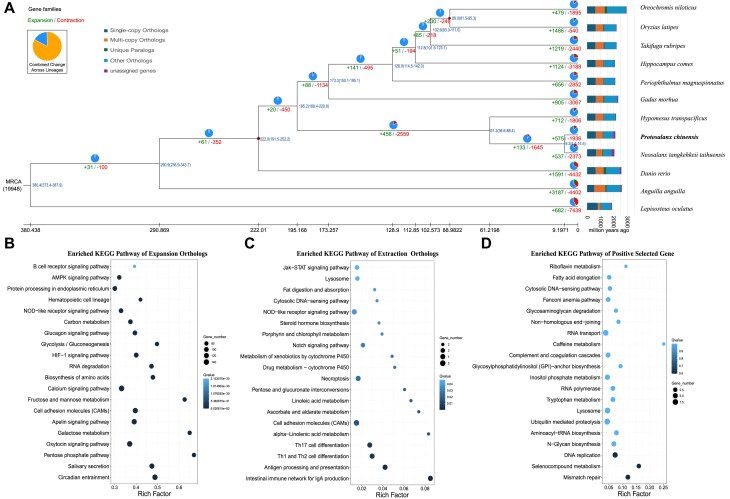
Genome evolution of *Protosalanx chinensis*. A: Phylogenetic relationship, divergence times, and gene families of *P. chinensis* and relevant actinopterygii. The gene families’ expansions (numbers in green) and contractions (numbers in red) are shown at individual lineages. Each node shows the estimated divergence times (blue numbers, Mya). Red dots indicate times taken from the TimeTree website. B: Enrichment analysis of expansion gene families of *P. chinensis* genome compared to other fishes, with the circles’ color representing the KEGG's statistical significance. The circle size represents the number of genes. C: Enrichment analysis of extraction gene families of *P. chinensis* genome compared to other fishes, with the circles’ color representing the KEGG's statistical significance. The circle size represents the number of genes. D: Enrichment analysis of positive selected gene of *P. chinensis* genome compared to other fishes, with the circles’ color representing the KEGG's statistical significance. The circle size represents the number of genes.

The comparative genomics analysis for *P. chinensis* and *Neosalanx taihuensis*, which exhibit the closest relationship, is being conducted. The number of identified Inversions, Translocations, and Duplications were 115,338, and 155 respectively (Supplementary Table S8 and Supplementary Fig. S4). The gene families were clustered among *P. chinensis* and eleven other fish species, namely *Oreochromis niloticus, Oryzias latipes, Takifugu rubripes, Hippocampus comes, Periophthalmus magnuspinnatus, Gadus morhua, Hypomesus transpacificus, Neosalanx tangkehkeii taihuensis, Danio rerio, Anguilla Anguilla*, and *Lepisosteus oculatus* to gain a comprehensive understanding of the phylogenetic analysis. A total of 262,179 genes (97.7% of 271,225) were clustered into 19,635 gene families in *P. chinensis* genome. Among these families, there were 19,949 gene families shared by all the fish species and 61 orthogroups containing 256 genes specific to *P. chinensis* (Supplementary Table S9). The identification and utilization of a total of 3,461 single-copy orthologous genes were employed for the construction of the phylogenetic tree (Fig. 2A). The phylogenetic tree revealed that *P. chinensis* is positioned within the same clade as *Neosalanx tangkehkeii taihuensis*, thereby providing additional evidence for intra-familial divergence within the Salangidae family. The divergence of the Salangidae family from *Hypomesus transpacificus* is estimated to have occurred approximately 61.2 million years ago (MYA). The divergence time between *P. chinensis* and *N. taihuensis* ranges from 4.7 to 14.5 million years ago (Fig. 2A), since their taxonomic classification as two distinct species.

After comparing with the other eleven fish genomes, a total of 575 orthogroups in *P. chinensis* were found to have expanded, while 1,936 orthogroups contracted (Fig. 2A). The statistical significance test (*P*<0.05) revealed the identification of 209 significantly expanded gene families and 259 contracted gene families. A total of 1716 expanded genes were involved, and these genes significantly enriched several pathways including Circadian entrainment, Salivary secretion, Calcium signaling pathway, and Oxytocin signaling pathway (Fig. 2B and Supplementary Table S10). The significantly expanded gene families can be involved in various Gene Ontology (GO) terms, including DNA integration (GO:0015074), methyltransferase activity (GO:0008168), oxidation-reduction process (GO:0055114), nucleic acid binding (GO:0003676) and GTPase activity (GO:0003924) (Supplementary Table S11). The significantly contracted gene families exhibited significant enrichment in the KEGG pathway, including Intestinal immune network for IgA production, Antigen processing and presentation, Th1 and Th2 cell differentiation, and Th17 cell differentiation (Fig. 2C and Supplementary Table S12). The selective pressure was analyzed using the Codeml program in PAML, resulting in the identification of a total of 391 positively selected gene (PSGs) that were significantly positively selected (*p*<0.05) in *P. chinensis* (Supplementary Table S13). These PSGs were functionally KEGG enriched in “Mismatch repair,” “Selenocompound metabolism” and “DNA replication” (*P*<0.05) (Fig. 2D and Supplementary Table S14). With GO annotation, four GO terms included binding (GO:0005488), DNA primase activity (GO:0003896), response to unfolded protein (GO:0006986) and 5-flap endonuclease activity (GO:0017108) can be significantly enriched (Supplementary Table S15).

### Whole genome resequencing and genetic variation

To comprehensive understanding the genomic footprint of diversity and adaptation, a total of 231 samples from seven different location including two native population (54) and five introduced population (177) were whole genome re-sequenced with an average of 14.33-fold genome coverage (Supplementary Fig. S5 and Supplementary Table S16). A total of 1.64 Tb clean data (average 7.1 Gb per individual) was generated, with an average coverage of 91.55% of the *P. chinensis* reference genome (Supplementary Table S17). The sample alignment rate ranges from 85.16% to 95.41%, with an average alignment rate of 93.27% (Supplementary Table S18).

The individual SNPs can reflect individual SNP differences between the target individual and reference genome. The high SNP number of the population represents native population originating from THL (Taihu Lake) and LHL (Lianhuan Lake), comprising average individual 786,827/784,184 SNP loci (Supplementary Tables S19 and S20). The average number of four introduced population is from 733,518 to 755,581 (Supplementary Tables S19 and S20). The other native population, originating from HZL (Hongze Lake) which is situated in the Huaihe River system and experiences frequent fishing activities. The average number of individual SNPs of HZL is 732,836. The population exhibited the lowest average number of SNPs per individual, indicating a significant loss of genetic diversity in this native population. The trend in Indel number is similar with that of the SNP number, except that HRCL has the lowest value with average 243,821 (Supplementary Tables S19 and S20). After minor allele frequency (MAF) and missing filtering, a total of 993,110 population single-nucleotide polymorphisms (SNPs) were identified for 231 samples.

### Population structure analysis

**Figure 3:**
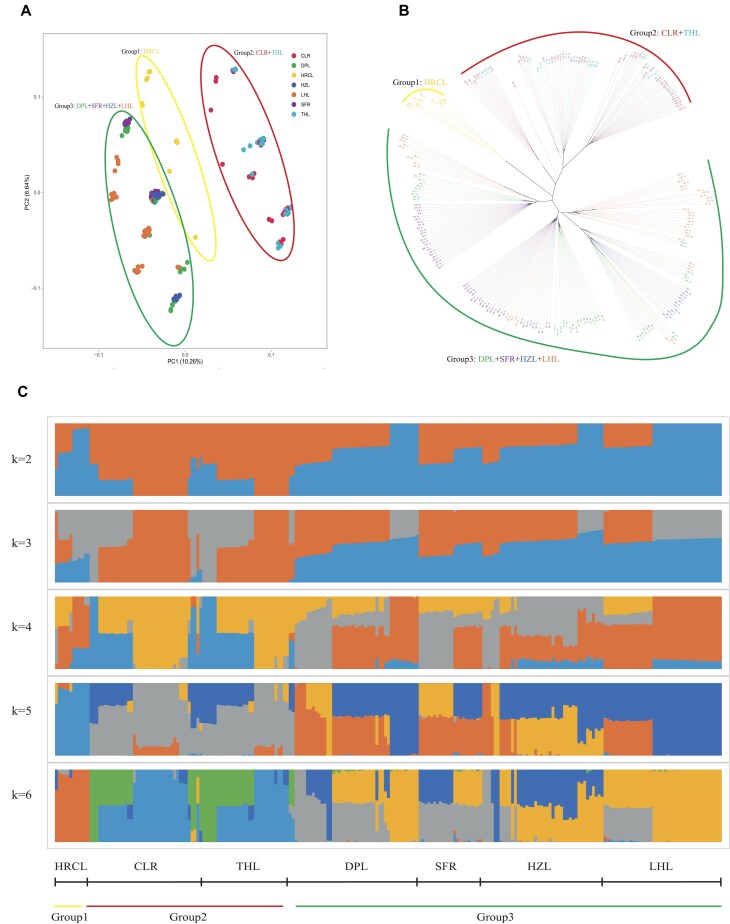
Population structure of 231 individuals in native and introduced *P. chinensis*. A: principal component analysis (PCA) of 231 individuals. B: Phylogenetic tree of SNPs. C: Population structure.

The evolutionary relationships were investigated by employing phylogenetic tree analysis, principal component analysis (PCA), and population structure analysis based on the population SNPs. The introduced population HRCL individuals can be clearly distinguished from the other six population in *P. chinensis* (Fig. 3). The 231 samples can be classified into three main groups based on the SNP set, namely group 1 (HRCL), group 2 (CLR and THL) and group 3 (DPL, SFR, HZL, and LHL) (Fig. 3A, B and C). These findings indicate that most of individuals exhibit an admixture structure in group 2 and group 3. The Group 1 (HRCL), situated in West Liaohe, exhibits high River Alkalinity levels (7.5 mg/L) and moderate Salinity levels (1.8 ‰). The extreme environmental conditions contribute to the distinct characteristics of fish within this group. The results of neigh-bour-joining (NJ) tree and PCA also indicated that HRCL individuals were significantly different from all other six populations.

### Genomic diversity and gene flow

**Figure 4:**
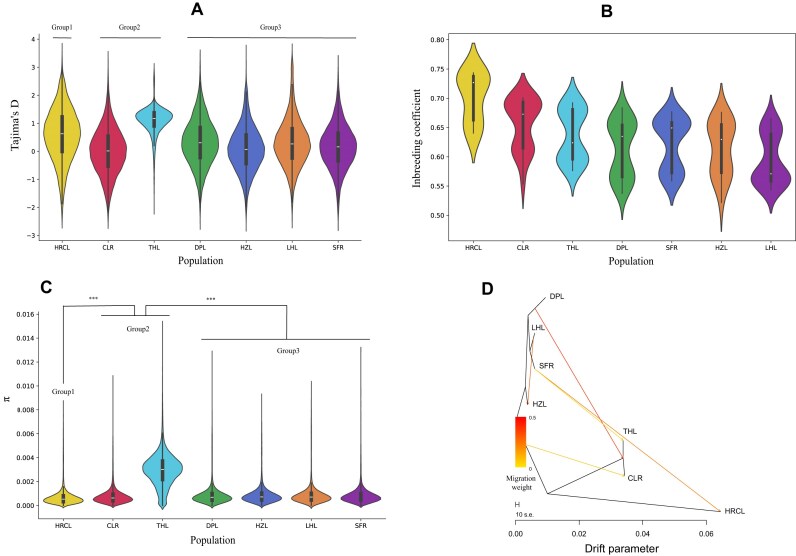
The population genetic statistics, inbreeding co-efficient and gene flow for different location in *Protosalanx chinensis*. A: Violin plot of Genome-wide Tajima's D. B: Inbreeding co-efficient. C: Nucleotide diversity (π). D: Gene flow with Tree-mix.

As previously study, the introduced process of alien colonizing populations frequently leads to the manifestation of apparent founder or genetic bottleneck effects [17]. The genetic diversity might be decreased as well as the positive Tajima's D, high LD patterns. The Tajima's D values for THL was 1.099, while the other groups exhibit values below 0.452 on average (Fig. 4A). The positive Tajima's D of native lake population and introduced population decreased significantly compared with THL (Fig. 4A). The LD decay also showed that a similar trend as the positive Tajima's D (Supplementary Fig. S6). The inbreeding coefficient, in contrast, exhibited an increase in introduced colonizing populations, thereby resulting in the occurrence of inbreeding depression [18]. The inbreeding coefficients of native lake groups were found to be 0.595 (0.544–0.665) for LHL and 0.615 (0.522–0.676) for HZL, while the introduced population exhibited higher values with HRCL at 0.702 (0.640–0.744), CLR at 0.656 (0.550–0.700), DPL at 0.613 (0.537–0684), SFR at 0.622 (0.559 -0.677), and THL at 0.637 (0.576 -0.692) (Fig. 4B). The high inbreeding coefficient were observed both in introduced populations and native lake population. The HRCL exhibited the highest inbreeding coefficient, which is consistent with its highest alkalinity level. The nucleotide diversity (π) average of THL is 0.002961, which is significantly higher than those of the other five groups (Fig. 4C). The π exhibited significant differences among group 1 to group 3. The native individuals of THL reside near the sea and belong to a lake population that may have experienced introgression from different ecotypes. The gene flow demonstrated the potential migration of SFR to THL, SFR to HRCL, and LHL to HZL (Fig. 4D). This gene flow is consistent with the patterns of river flow and historical introductions.

### Selective sweep analysis

**Figure 5:**
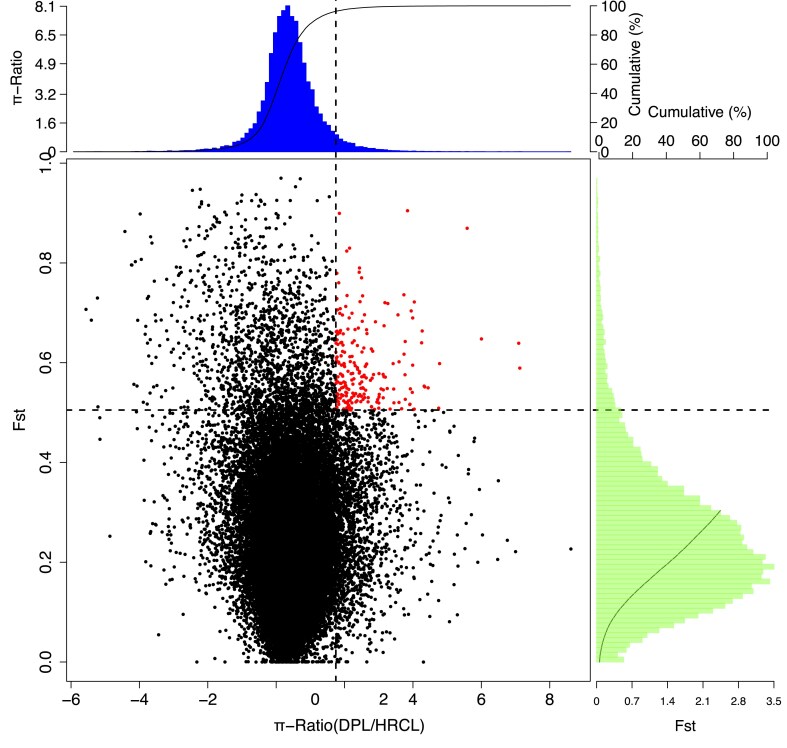
Significant selected regions (red) analysis based on F_ST_ and π.

To reveal the genomic signatures associated with adaptation to varying levels of alkalinity and salinity in populations, the selective sweep analysis was performed. The HRCL (highest alkalinity at 7.5 mg/L) and DPL (lowest alkalinity at 0.89 mg/L) were compared for alkalinity adaption, while DPL (lowest salinity at 0.1 mg/L) was utilized to identify genomic imprints in salinity adaption. The loci that differentiate between DPL and HRCL (Fig. 5) may be under strong selection with respect to alkalinity, as indicated by the combination of Fst and π values in the upper 5% tail of the distribution. The GO enrichment analysis revealed that the selected region harbors a majority of 51 genes associated with integral component of membrane (GO:0016021), along with 28 genes linked to zinc ion binding (GO:0008270) and an additional 28 genes involved in calcium ion binding (GO:0005509) (Supplementary Table S21). Both sodium channel activity (GO:0005272) and sodium ion transport (GO:0006814) were enriched with GO enrichment analysis. We integrated the sliding window results of three methods (Fst, π and XP-CLR), we identified ∼2.13 Mb putative regions, including 215 candidate genes, which might be associated with alkalinity or adaption. The region (Chr12: ∼5Mb) showed that highly diversity including gene associated sodium ion transport (Fig. 5).

## Discussion

The newly assembled genome in this study belongs to the T2T level with gap-free contigs (the contig N50 value is approximately 14 Mb), which is a significant improvement compared to previous genome assemblies, such as the contig N50 is 17.2 Kb in Liu et al. 2017 [2], 103 Kb in Zhang et al. 2020 [3] and 530 Kb in Zhou et al. 2023 [12]. The presence of completely intact telomere repeat monomer sequences in the T2T genome highlights its exceptional quality, rendering it a valuable resource for studies on telomeres, centromeres, and satellite DNA. The genome annotation with high confidence in quality implies that the gene set can serve as a good reference.

The comparative genomics analysis revealed distinct gene characteristics in *P. chinensis*, which were attributed to the expansion or contraction of gene families. The bony fishes, *P. chinensis*, possess the most genes associated with bone formation; however, certain calcium-binding phosphoproteins have been either lost or rendered pseudogenes in previous studies [3]. In this study, the expanded calcium signaling pathway maybe related with the bone formation. The presence of an expanded gene associated with circadian entrainment suggests that *P. chinensis* may exhibit a high sensitivity to photoperiod. This circadian rhythm influences various physiological characteristics, including growth, feeding, and reproduction. The similar results also had been found in humpback grouper [19]. Another specific features for *P. chinensis*, the immune system or sensitive to stress for example air exposure stress [3, 12]. The immune system in fish larvae exhibits limited resilience [20]. Another specific feature of *P. chinensis* is its immune system which can be related with susceptibility to stressors, such as air exposure stress [3, 12]. The expanded genes' GO terms, including methyltransferase activity and oxidation-reduction process, may be associated with its broad environmental tolerance [3]. The contracted genes immune-related families including NOD-like receptor signaling, T cell receptor signaling pathway and Toll-like receptor signaling pathway was consistent with the previously studies [3]. The other genes immune-related families including Intestinal immune network for IgA production which related with host-microbe interaction [21], antigen processing and presentation which are the cornerstones of adaptive immunity [22]. The gene under positive selection is enriched in the biological processes of “Mismatch repair” and “DNA replication,” which are evolutionarily conserved for maintaining genome stability [23]. It maybe suggested that *P. chinensis* underwent selection pressure in DNA repair during adaptive evolution.

To reveal the genetic diversity, all the 7 sample sites with 231 individuals had been whole genome resequencing. The native population (THL) exhibits the higher diversity, as indicated by the high number of single nucleotide polymorphisms (SNPs) observed (average 0.784 M), which aligns with the general trend of wild samples typically displaying greater diversity [24]. The samples of LHL, representing the introduced population from a lake, exhibit an average of 0.787 million SNPs, which is significantly higher compared to the other group population. However, The HZL which is a native population (lake) but introduced population was applied in 28 December 2020 from LHL and SFR, which maybe lead to the samples from both native lake samples and Introduced population. The SNP number of HZL showed similar with other introduced samples (from 0.733 to 0.755 M). The introduction resulted in a significant decrease in SNP diversity, as observed through comparative analysis. This phenomenon was also evident in the values of Tajima's D and nucleotide diversity (π). The THL exhibited significant genetic diversity, while other introduced populations experienced a loss of diversity, indicating the occurrence of inbreeding depression among these colonizing populations [18]. The reduction in genetic diversity may have contributed to the post-introduction failure of *P. chinensis* in China [6].

The population structure analysis revealed a clear distinction between HRCL and other samples, which contradicts the findings of previous studies that suggested THL is different with other groups [6]. This discrepancy may be attributed to variations in sample size or different sampling locations. Subsequently, HRCL clearly formed a distinct cluster characterized by the highest alkalinity. This suggests that extreme local environmental adaptation has led to population divergence. The local sheep breeds in Xinjiang also demonstrate a wide range of environmental adaptability, reflecting a similar phenomenon [25]. The samples of CLR and THL can be classified as group 2, suggesting the possible introduction of CLR from THL, which is commonly considered one of the potential origin sites for introduction. The gene flow (treemix) analysis revealed that there was a potential gene flow of SFR to THL and HRCL, which aligns with the introduced event to Taihu Lake and Chuole reservoir and different with the natural migration [26]. The potential migration of LHL to HZL also had been found in treemix results which can be explain why the low genetic diversity of HZL and the nearly relation with LHL. The HZL (Hongze Lake) fishing management office news reported a significant enhancement and release event that took place on December 12, 2020 for HZL, where most of fish spawn originates from LHL. With the accuracy introduced record, it showed that the treemix results can be as evidence for gene flow existed among sampling locations [27]. The samples of DPL, SFR, HZL, and LHL were classified as group 3, indicating potential repeated introduction among these regions, suggesting that the samples can be mixed. The lack of detailed historical records makes it extremely challenging to deduce the clearly delineated process. However, the results of genetic diversity or gene flow suggest a potential relationship or introgression occurring among populations in different locations.

The alkalinity and salinity are the primary factors influencing the fish water environment. Here, the genomic signatures associated with selection pressures were performed. The differentiated loci with GO terms enriched in integral component of membrane, zinc ion binding and calcium ion binding.

The integral component of the membrane facilitates the active transport of ions against their electrochemical gradient [28]. The candidate genes exhibiting zinc ion binding and calcium ion binding provide further evidence supporting the hypothesis that the transformation habit is influenced by alkalinity and salinity. The phenomenon of salinization has been observed globally, resulting in local adaptations [29]. The physiological demands imposed by alternative salinity environments exert significant and contrasting pressures on osmoregulatory systems, thereby driving natural selection to act upon the mechanisms enabling fish to perceive and adapt to salinity fluctuations [30]. The alkalinity or salinity transitions associated with osmotic environments give rise to adaptations in osmoregulatory physiology and a complex suite of integrated traits [31]. Gene expression related Na^+^/K^+^-ATPase with associated with water and ion exchange accompany salinity tolerance [32]. Migration and introduction may subject *P. chinensis* to osmoregulatory stress in various populations. Reduced genetic and purging selection had been found in introduced highest alkalinity population.

## Conclusion

Our study presents a novel T2T reference genome of *P. chinensis*, which significantly enhances the contiguity, accuracy, and completeness. The assembled genome size is 380.76 Mb, consisting of one contig per chromosome. The repeat sequences account for 137.79 Mb (35.9%) and a total of 21,073 protein-coding genes have been annotated. Whole genome resequencing of 231 samples from seven different location reveals the genetic variation and population genetics. The HRCL can be distinctly differentiated from the other six different geographical samples, which can be categorized into another two primary groups. The high inbreeding coefficients were observed in both introduced populations and the native lake population. The gene flow is consistent with the patterns of river flow and historical introductions. The selection sweep analysis encompasses GO terms associated with integral components of the membrane, zinc ion binding, and calcium ion binding, which are relevant to the adaptation to alkalinity and salinity.

## Methods

### Sample collection and extraction

Female/male fish were harvested from Taihu Lake in China. Fish muscle was collected for genomic DNA and Hi-C, respectively. The muscle tissue was collected from a total of 231 individuals sampled across 7 geographic sites including 2 native populations (Taihu Lake is located in the Yangtze River system: THL, n=32; Hongze Lake is located in Huaihe River system: HZL, n=22) and five introduced populations (Dongping Lake is located in Yellow River system: DPL, n=43; Shuifeng Reservoir is located Yalu River system: SFR, n=41; Lianhuan Lake is located in Nenjiang River system: LHL, n=42; Harqin Zuoyi Lake is located in West Liaohe River system: HRCL, n=12; Chuole reservoir is located in Nenjiang River system: CLR, n=39) for subsequent next generation sequencing analysis. The muscle tissue was collected, rapidly cryopreserved in liquid nitrogen, and stored at -80°C for subsequent DNA sequencing. The genomic DNA was extracted using the DNeasy Blood and Tissue kit (Qiagen). The transcriptome RNA was extracted using TRIzol™ (Invitrogen, Carlsbad, CA).

### Library construction and sequencing

The Telomere-to-telomere gap-free genome can be assembled using two types of long-read technologies, namely PacBio HiFi and Nanopore ultra-long library. The PacBio HiFi library (∼20 kb insert size) was constructed using the QIAGEN Blood & Cell Culture DNA Midi Kit according to the manufacturer's instructions (QIAGEN, Hilden, Germany). The ultra-long nanopore library was treated using Ligation sequencing 1D kit (SQK-LSK109). The RNA libraries were constructed using TRIzol Total RNA Isolation Kit (Takara, USA). The PacBio HiFi library was sequenced using the CCS mode on the PacBio Sequel II SMRT cell (RRID:SCR_017990). The ultra-long nanopore library was sequenced on the Promethion platform. For re-sequencing data, the short insert size libraries (∼350 bp) were performed following MGI manufacturer's recommendations and PE 150 reads were sequenced on DNB-seq T7 platform (RRID: SCR_024847). The utilization of Hi-C data is valuable for the purpose of chromosomal genome anchoring. The fish muscle tissues were treated with 1% formaldehyde at room temperature for 10–30 minutes to crosslink proteins that interact with chromatin. Then, the Mbo I restriction enzyme (NEB, Ipswich, USA) was subsequently employed for digestion to construct the Hi-C library [33]. The Hi-C library and RNA libraries were sequenced using the PE (Paired-end) 150 bp protocol on the DNB-seq T7 platform.

### Data processed and genome assembly

The HiFi data were processed using the SMRT Link (v8.0.0) CCS algorithm with parameters “–minPasses 3 –minPredictedAccuracy 0.99 –minLength 500.” The HiFi data were processed using the SMRT Link (v8.0.0) CCS algorithm [34], employing parameters “–minPasses 3 –minPredictedAccuracy 0.99 –minLength 500.” The clean HiFi reads were assembled using Hifiasm (RRID:SCR_021069) (v0.16.1-r375) [35] with default parameters and the redundant sequences were processed using Purge-Haplotigs with the following parameters: “-j 80 -s 80 -a 75.” The assembled contig were subsequently anchored to chromosomes using Juicer (v1.5) [36] and 3D-DNA (v180922) (RRID:SCR_017227) pipeline [37]. The errors were processed and curated using Juicebox (v1.11.08) [36]. Nanopore ultralong reads were processed by filtering out sequences shorter than 5 kb or with a quality value below 7. Those reads were corrected firstly using Necat software (v 20200119) [38]. The corrected ultralong reads were used to fill the gaps based on chromosome level genome using LR_Gapcloser (v1.0) (RRID:SCR_017227) [39] and TGSgapcloser (v 1.0.1) [40]. The telomere-to-telomere gap-free genome was ultimately generated. The centromeres and telomeres were isolated using the quartet (v 1.1.1) (RRID:SCR_025258) method.

### Genome annotation

The genome annotation contained the identification of repetitive elements, prediction of protein-coding genes, and functional annotation. The identification of long terminal repeats and repetitive elements was performed using LTR-FINDER (v1.0.7) (RRID:SCR_015247) [41] and RepeatModeler (v1.0.4) (RRID:SCR_015027) [42], respectively, employing their default parameters. The above results were utilized to generate a *de novo* repeat sequence library. The RepeatMasker software (v4.0.7) (RRID:SCR_012954) [43] was utilized to identify low-complexity sequences and interspersed repeats based on the provided library. In homolog-based prediction, the RepeatProteinMasker (v4.0.7) and RepeatMasker (v4.0.7) [43] tools were utilized for the identification of protein transposable elements (TEs) and DNA sequences based on the Repbase database (RRID:SCR_021169) [44]. The identification of tandem repeats was performed using Tandem Repeat Finder (v4.10.0) (RRID:SCR_022065) [45].

The prediction of genes involved a comprehensive approach that included homology-based, ab initio, and transcriptome-based methods. The RNA-seq data were aligned to the *P. chinensis* genome using Hisat2 (v2.1.0) (RRID:SCR_015530) [46] to delineate potential exon regions and splice junctions. Subsequently, StringTie (v1.3.5) (RRID:SCR_016323) [47] was utilized for assembling the mapped reads into gene models, which were further validated via PASA (v2.5.2) (RRID:SCR_014656) [48]. Candidate coding regions were identified using TransDecoder (v5.5.0) (RRID:SCR_017647) [49 ]. For homology-based annotation, genome assembly and gene annotation files from four actinopterygii species (*Danio rerio, Oryzias latipe*s, *Protosalanx hyalocranius*, and *Salmo salar*) were retrieved from the NCBI database. Leveraging this RNA-seq and homologous data, homology-like coding sequences were predicted with GeMoMa (v1.8) (RRID:SCR_017646) [50]. A dataset of 1200 high-quality coding genes was employed to train predictors, utilizing August (v3.2.1) and SNAP (v2006-07-28) (RRID:SCR_007936), followed by ab initio prediction. Integration of protein-coding genes forecasted by the mentioned strategies was accomplished using the EVidenceModeler (EVM) pipeline (v1.1.1) (RRID:SCR_014659) [48].

### Comparative genomic and Gene Family Analysis

The chromosomal structural variations were identified between *P. chinensis* and *Neosalanx taihuensis* using SyRI (RRID:SCR_023008) [51]. OrthoFinder (v2.3.11) (RRID:SCR_017118) [52] was used for the clustering of protein-coding genes, and single-copy orthologous genes families (1:1:1) were aligned using MAFFT (v7.3) (RRID:SCR_011811) [53]. Subsequently, a maximum-likelihood phylogenetic tree was constructed with PhyML (v3.3) (RRID:SCR_014629) [54] employing the HKY85 model. Consistent phylogeny with a previous study was demonstrated, with all branches showing 100/100 bootstrap support. The species divergence time estimation was conducted using MCMCTREE in PAML (v4.9) (RRID:SCR_025348) [55], calibrated with four divergence time points from TimeTree (RRID:SCR_021162): (a) *Lepisosteus oculatus* and *Anguilla anguilla* (373.4–387.9 MYA), (b) *D. rerio* and *Gadus morhua* (191.5–252.2 MYA) and (c) *Oreochromis niloticus* and *O. latipes* (81.5–95.3 MYA). Homologs were searched using protein sequences of *P. chinensis* and other 11 published Actinopterygii species. Expansion and contraction analyses in the branch of finless porpoises were performed based on the gene families clustered by OrthoFinder, utilizing the CAFÉ (v4.0) software (RRID:SCR_005983). Gains and losses of gene families in a user-specified phylogeny were studied employing random birth and death models. The estimation of the global parameter λ, describing both the gene birth (λ) and death (μ = −λ) rate for gene families in all branches of the tree, was conducted using maximum likelihood. Subsequently, a P-value was calculated for each gene family, with a threshold of P-value ≤ 0.01 defining a “significantly expanded and contracted gene family.” KEGG and GO enrichment analyses were carried out among these significantly expanded and contracted gene families. Single copy orthologs were identified using protein sequences of *P. chinensis* and other 11 published Actinopterygii species. Ka/Ks ratios for these single copy orthologs were calculated through the following steps. Initially, a global alignment among these single copy orthologs was performed using PRANK, followed by filtering the alignment with Gblocks. Finally, Ka/Ks ratios on different branches were calculated using Codeml in the PAML package with the free-ratio model. Genes displaying Ka/Ks values higher than 1 along the branch leading to the finless porpoise were subjected to reanalysis using the codon-based branch site tests implemented in PAML (RRID:SCR_014932). The branch site model, allowing ω to vary both among sites in the protein and across branches, was utilized to detect episodic positive selection.

### Re-sequencing data processed and alignment

The short-reads used for whole genome re-sequencing was processed using SOAPnuke(Version:v2.2.6) [56] with parameters “-n 0.001 -l 10 -q 0.5 -Q 2.” The clean processed reads were mapped onto the new assembled reference *P. chinensis* genomes using BWA-MEM 0.7.17 [57]. The alignment output in SAM format was converted to BAM format using samtools (v1.3) [58]. Subsequently, the BAM file was indexed, sorted, and marked for duplicates. The genetic variation included SNPs and Indels was called using GATK 5 (Version:V4.1.2.0) [59] with the module of HaplotypeCaller. All the individual GVCF files were combined and merged into one file. The annotation of SNPs and Indel was performed using ANNOVAR (v2015) [60]. The SNPs were filtered with the parameter “QD < 2.0 || FS > 60.0 || MQ <40.0 || MQRankSum < -12.5 || ReadPosRankSum < -8.0.” The Indels were filtered with “QD < 2.0 || FS > 200.0 || SOR > 10.0 || MQRankSum < -12.5 || ReadPosRankSum < -8.0.” The subsequent analysis utilized SNPs with a SNP quality >30 and site frequency spectrum (minor allele frequency MAF≥ 0.05 and missing rate ≤ 0.1) to ensure precise results.

### Population genetics analysis

The SNP-based phylogenetic tree was constructed using Phylip v3.696 (RRID:SCR_006244) [61] and the tree was visualized using iTOL (Version:6.8.1) [62]. The admixture software (Version: 1.3.0) [63] is utilized for the estimation of genetic structure in all individuals. The principal component analysis and kinship analysis were conducted using the GCTA software (Version: 1.94.0) [64] for all samples. The top three levels, namely PC1, PC2, and PC3, are visualized in the PCA result. To detect the diversity between sub-populations, the pairwise estimates of differentiation Fst and nucleotide diversity (π) was determined using VCFtools v0.1.15 (RRID:SCR_001235) [65] using a sliding window. The window size was set to 10 kb with a step size of 5 kb. The XP-CLR test was performed using XP-CLR v1.1.2 with default parameters and window size 10 kb and step size 5 kb [66]. The linkage disequilibrium (LD) was computed by calculating the pairwise correlation coefficients (r2) between genotypes using PopLDdecay-3.42 with parameters (-MaxDist 1000 -MAF 0.05 -Miss 0.1) [67].

## Additional Files

**Supplementary Figure S1:** Divergence distribution of repetitive elements in *Protosalanx chinensis* genome.

**Supplementary Figure S2:** Venn diagram of gene annotation based on 5 databases (NR, InterPro, KEGG, SwissProt, and KOG).

**Supplementary Figure S3:** The gene features distribution of *Protosalanx chinensis* and the other 4 fish species.

**Supplementary Figure S4:** The chromosomal structural variations identified between *Protosalanx chinensis* and *Neosalanx taihuensis* species.

**Supplementary Figure S5:** The geographic distribution of 7 sampling sites.

**Supplementary Figure S6:** The LD decay of 7 native and introduced populations.

**Supplementary Table S1:** Summary of the sequencing data obtained for WGS libraries, PacBio HIFI library, ONT libraries, and Hi-C libraries.

**Supplementary Table S2:** The statistics of telomere repeat for the *P. chinensis* genome.

**Supplementary Table S3:** Summary of transposon element families in the *P. chinensis* genome based on various methods.

**Supplementary Table S4:** Statistics of classified repeat in the *P.chinensis* genome.

**Supplementary Table S5:** Summary of predicted protein-coding genes in the *P. chinensis* genome.

**Supplementary Table S6:** The evidence supporting gene models of the *P. chinensis* genome.

**Supplementary Table S7:** Statistics of genes with functional classification by various methods in the *P. chinensis* genome.

**Supplementary Table S8:** The statistics of identified chromosomal structural variations between *P. chinensis* and *Neosalanx taihuensis*.

**Supplementary Table S9:** Gene family clustered.

**Supplementary Table S10:** KEGG enrichment of expanded gene families in the *P. chinensis* genome.

**Supplementary Table S11:** GO enrichment of expanded gene families in the *P. chinensis* genome.

**Supplementary Table S12:** KEGG enrichment of extracted gene families in the *P. chinensis* genome.

**Supplementary Table S13:** Positively selected genes (PSGs) in the *P. chinensis* genome.

**Supplementary Table S14:** KEGG enrichment of positively selected gene families in the *P. chinensis* genome.

**Supplementary Table S15:** GO enrichment of positively selected gene families in the *P. chinensis* genome.

**Supplementary Table S16:** The detailed information of 231 samples collected from 7 different locations.

**Supplementary Table S17:** The summary of sequencing data for 231 samples.

**Supplementary Table S18:** The summary of alignment for 231 samples.

**Supplementary Table S19:** The genetic variation including SNP and indel statistics in 231 samples.

**Supplementary Table S20:** The genetic variation statistics, including average SNPs and indels, are compared between different populations and the reference genome.

**Supplementary Table 21:** The GO enrichment of the differentiated region between DPL and HRCL.

giaf067_Supplemental_Files

## Abbreviations

BUSCO: Benchmarking Universal Single-Copy Orthologs; CDS: coding sequences; GO: Gene Ontology; KEGG: Kyoto Encyclopedia of Genes and Genomes; LD: linkage disequilibrium; MAF: minor allele frequency; MYA: million years ago; NJ: neighbor-joining; PCA: principal component analysis; PSG: positively selected gene; QV: quality value; SNP: single-nucleotide polymorphism; T2T: telomere-to-telomere; TE: transposable element.

## Data Availability

The *P. chinensis* whole genome sequencing and assembly are deposited to NCBI databases under accession number PRJNA1065125. The assembly and annotation files are available at Figshare [68]. All additional supporting data are available in the *GigaScience* repository, GigaDB [69].
