# Supplementary material for: Telomere-to-telomere genome and resequencing of 231 individuals reveal evolution, genomic footprints in Asian icefish, Protosalanx chinensis
Source: Gigascience. 2025 Jul 17;14:giaf067. doi: 10.1093/gigascience/giaf067 (PMC12266836; doi:10.1093/gigascience/giaf067)
Supplement: giaf067_Supplemental_Files [file giaf067_Supplemental_Files.zip › Supplementary Figures.docx]

Supplementary Materials

Supplementary Figure S1. Divergence distribution of repetitive elements in *Protosalanx chinensis*

genome.

Supplementary Figure S2. Venn diagram of gene annotation based on 5 databases (NR, InterPro, KEGG, SwissProt, and KOG).

Supplementary Figure S3. The gene features distribution of *Protosalanx chinensis* and the other four fish species.

Supplementary Figure S4. The chromosomal structural variations identified between *Protosalanx chinensis* and *Neosalanx taihuensis* species.

Supplementary Figure S5. The geographic distribution of 7 sampling sites.

Supplementary Figure S6. The LD decay of 7 native and introduced populations.


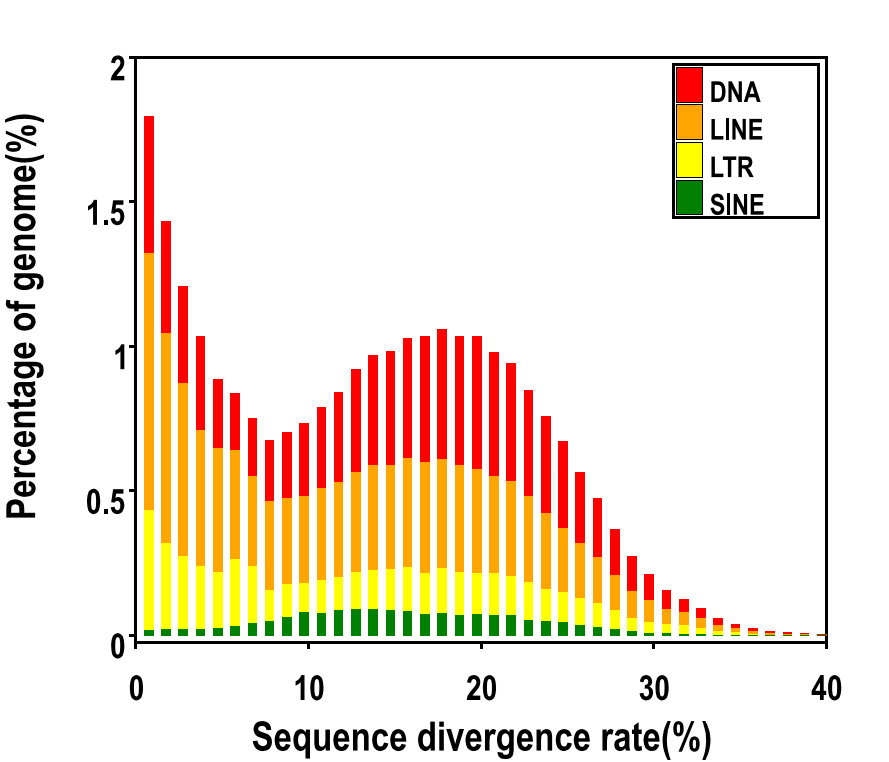


Supplementary Figure S1. Divergence distribution of repetitive elements in Protosalanx chinensis

genome.


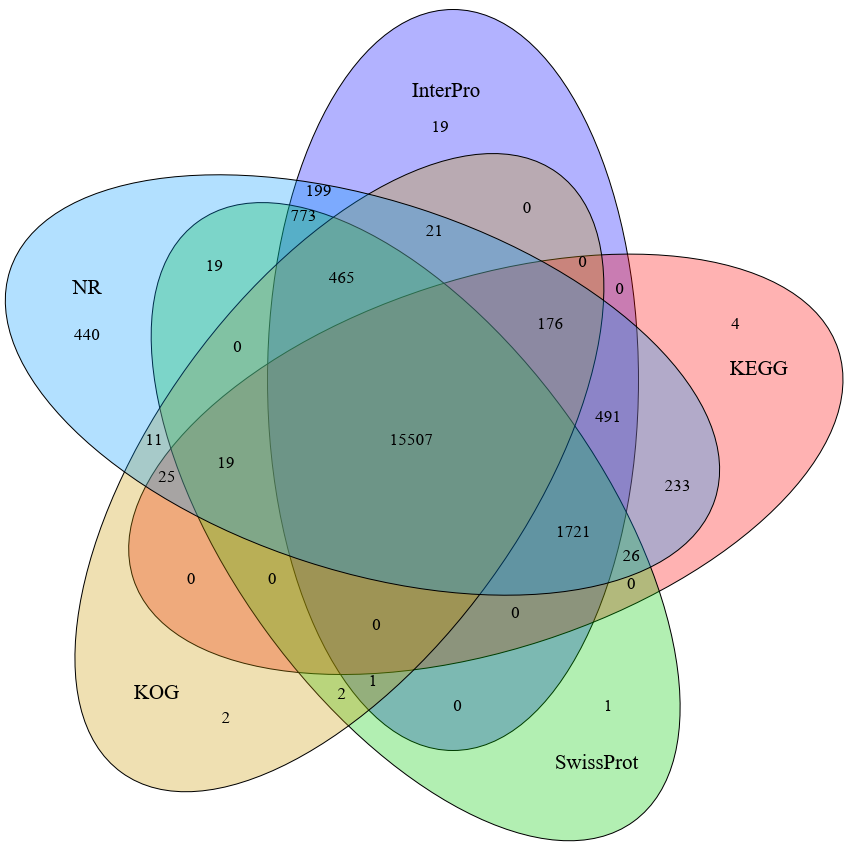


Supplementary Figure S2. Venn diagram of gene annotation based on 5 databases (NR, InterPro, KEGG, SwissProt, and KOG).


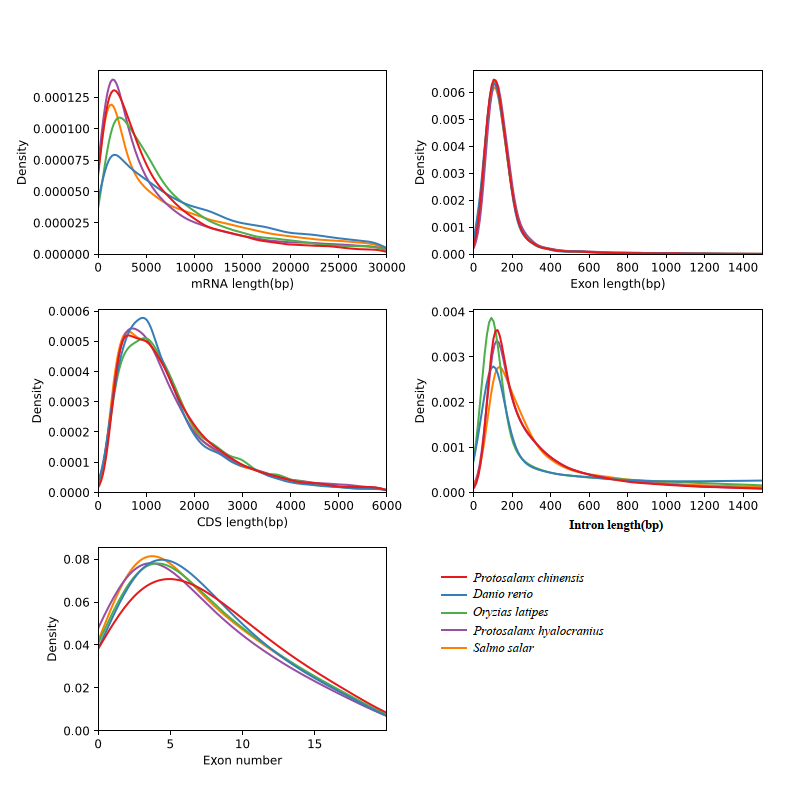


Supplementary Figure S3. The gene features distribution of Protosalanx chinensis and the other four fish species.


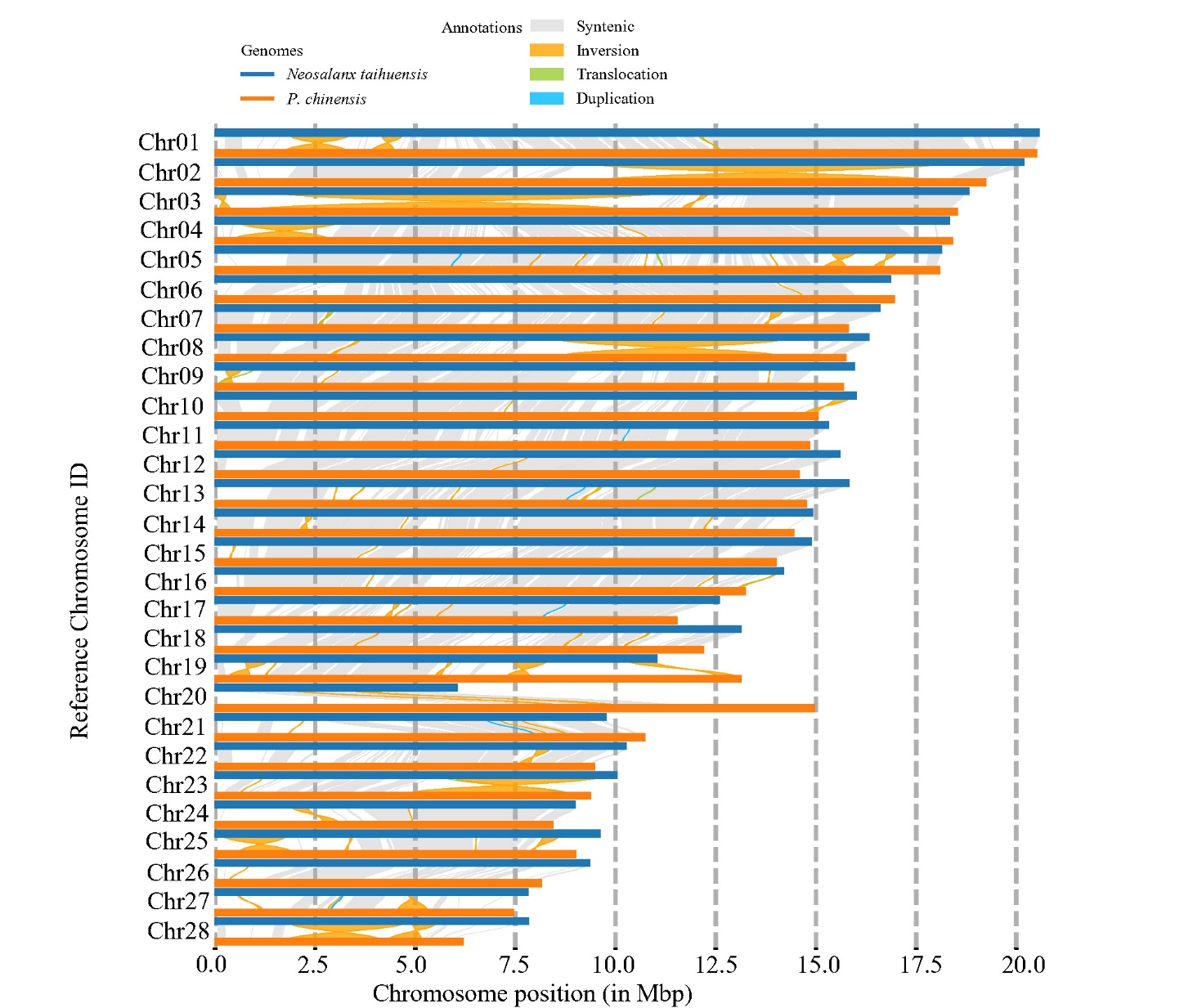


Supplementary Figure S4. The chromosomal structural variations identified between *Protosalanx chinensis* and *Neosalanx taihuensis* species.


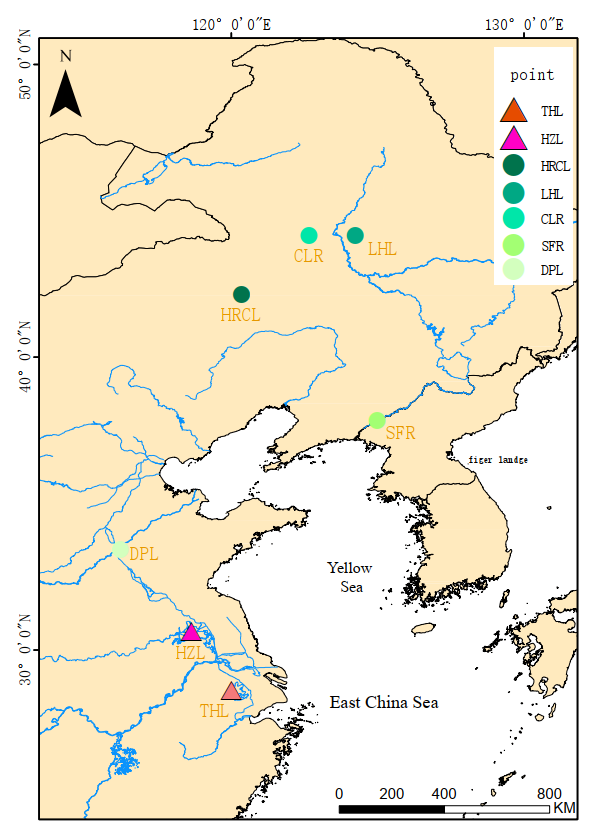


Supplementary Figure S5. The geographic distribution of 7 sampling sites.


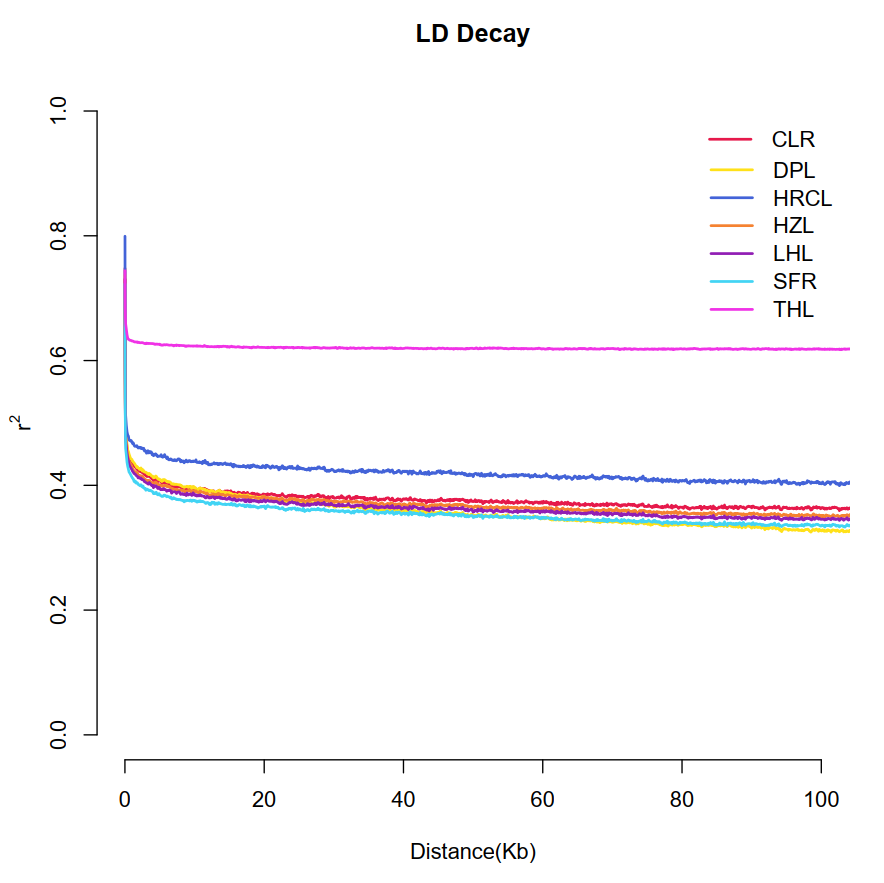


Supplementary Figure S6. The LD decay of 7 native and introduced populations.
